# Increase of Deep Intraepithelial Lymphocytes in the Oxyntic Mucosa of Patients With Potential and Overt Autoimmune Gastritis

**DOI:** 10.3389/fimmu.2022.866167

**Published:** 2022-05-04

**Authors:** Marco Vincenzo Lenti, Alessandro Vanoli, Emanuela Miceli, Giovanni Arpa, Michele Di Stefano, Simone Soriano, Francesca Capuano, Antonella Gentile, Nicola Aronico, Luigi Coppola, Alessandra Pasini, Ombretta Luinetti, Aurelio Mauro, Marco Paulli, Catherine Klersy, Gino Roberto Corazza, Antonio Di Sabatino

**Affiliations:** ^1^Department of Internal Medicine, Istituto di Ricovero e Cura a Carattere Scientifico (IRCCS) Fondazione Policlinico San Matteo, University of Pavia, Pavia, Italy; ^2^Department of Pathology, Istituto di Ricovero e Cura a Carattere Scientifico (IRCCS) Fondazione Policlinico San Matteo, University of Pavia, Pavia, Italy; ^3^Unit of Clinical Epidemiology and Biometry, Istituto di Ricovero e Cura a Carattere Scientifico (IRCCS) Fondazione Policlinico San Matteo, University of Pavia, Pavia, Italy

**Keywords:** anti-parietal cell antibody, atrophy, autoimmune gastritis, CD3, intraepithelial lymphocyte

## Abstract

Pathological correlates of potential autoimmune gastritis (AIG), defined by anti-parietal cell antibody (PCA) positivity in the absence of gastric atrophy, have never been described. We herein aimed to assess intraepithelial lymphocyte (IEL) infiltration in gastric corpus of AIG patients. From 2000 to 2021, among 53 potential AIG patients, we focused on nine (median age 61 years, IQR 53-82; four females) who subsequently developed overt AIG. IEL infiltration of the oxyntic mucosa was assessed before and after developing overt AIG by measuring deep and superficial CD3+ IEL. AIG patients with different degrees of corpus atrophy, healthy controls (HC), active H. pylori gastritis, celiac disease (CD), and Hashimoto’s thyroiditis patients were included as controls. Of note, deep, but not superficial, CD3+ IEL count was higher (p<0.001) in potential AIG compared to HC and H. pylori gastritis. Deep CD3+ IEL infiltration did not change before or after the evolution into atrophy (median 9.6, IQR 8.8-12.4, vs 11.3, IQR 9.4-12.9). No difference was found in deep CD3+ IEL infiltration among potential, mild, and severe AIG, and compared to Hashimoto’s thyroiditis or CD. A deep CD3+ IEL cut-off of >7/100 epithelial cells allowed discrimination of any AIG stage and severity (AUC=0.842). We conclude that an increased deep CD3+ IEL infiltration of the oxyntic mucosa could represent a marker of potential AIG. Prospective studies including a larger number of potential AIG patients are needed.

## Introduction

Autoimmune gastritis (AIG) is a slowly progressive, organ-specific disease characterized by the immune-mediated destruction of the gastric parietal cells, which results in hypo-achlorhydria and intrinsic factor deficiency, determining over time iron and vitamin B12 malabsorption (1, 2). AIG is also considered a pre-neoplastic condition, predisposing to the development of both gastric adenocarcinoma and type 1 neuroendocrine tumor (1, 3). The diagnosis of AIG can be difficult to make due to its proteiform manifestations (4, 5), and its possible association with many other autoimmune diseases (6–9). For these reasons, a significant diagnostic delay in AIG has been reported (10), and this could lead to complications (11) and to reduced physical functioning (12). All these considerations support the necessity of a reliable and early diagnosis based on screening with laboratory tests and serum AIG autoantibodies, including anti-parietal cell (PCA) and anti-intrinsic factor antibodies, along with histopathological confirmation (corpus-restricted atrophic gastritis which spares the antrum) (1, 13). In this regard, we have recently described a group of patients displaying PCA positivity with an architecturally normal gastric mucosa, who have been followed up for a median time of 8 years, and who developed mild-to-severe corpus atrophy (i.e., overt AIG) (11). Hence, we categorized these PCA-positive patients as being affected by a pre-atrophic phase of AIG, which was defined “potential AIG”, bringing this concept from celiac disease (CD). Nonetheless, early histopathological markers of potential AIG have not yet been described, although it is crucial to identify and follow up with these patients, so to prevent potentially irreversible complications. Also, these histopathological features could be a useful marker for raising the suspicion of AIG in patients undergoing upper gastrointestinal endoscopy (UGE) with collection of gastric biopsies. Of note, T cells infiltrating the gastric mucosa and the oxyntic epithelial layer have been shown to be key players in determining parietal cell apoptosis in AIG, possibly through perforin/granzyme and FAS/FAS ligand pathways, and, over time, inducing metaplastic and atrophic changes (14, 15). Similar to what is known for CD, in which the association of increased intraepithelial lymphocyte (IEL) infiltration of the duodenum and serum CD-specific autoantibodies define a pre-atrophic phase of CD, i.e., potential CD (16), we hypothesized that increased gastric corpus IEL in PCA-positive patients, with no histologic features of AIG, could be a marker for identifying potential AIG patients. On this basis, we sought to evaluate CD3+ IEL infiltrating both deep and superficial oxyntic glands in a group of patients with potential AIG, before and after the occurrence of overt AIG in comparison to other digestive (active H. pylori gastritis, active CD) and non-digestive (Hashimoto’s thyroiditis) disorders known to be associated with increased gastric IEL infiltration (17) in order to determine whether this histologic feature could be indicative of evolution of AIG into gastric corpus atrophic lesions.

## Materials and Methods

### Patients

This is a single center (Fondazione IRCCS Policlinico San Matteo, University of Pavia) prospective study. All potential AIG patients have been enrolled from January 2000 to June 2021 at the Department of Internal Medicine, in a gastroenterology outpatient clinic dedicated to the diagnosis and treatment of AIG. We enrolled patients undergoing UGE because of serum PCA positivity. Gastric biopsies were collected according to the Sydney-Houston protocol (18), in order to ascertain the absence of intestinal metaplasia or atrophy, deep or full-thickness lamina propria lymphoplasmacytic infiltrates, or micronodular enterochromaffin-like cell (ECL) hyperplasia. These patients were categorized as having potential AIG. Serum PCA were assessed in all patients through either an immunofluorescence kit with titers equal to, or greater than, 1:40 considered as positive, or an enzyme-linked immunosorbent assay kit (positive if >10 U/mL). PCA were tested in patients in whom AIG was suspected by the physician (ADS, EM, MVL) (4, 10) on the basis of vitamin B12 and/or iron deficiency and its consequences (e.g., pernicious, iron deficiency, or dimorphic anemia), dyspepsia, autoimmunity (i.e., autoimmune thyreopaties, vitiligo, type 1 diabetes), infertility and/or recurrent miscarriage, neurological alterations (i.e., paresthesia, ataxia, memory loss, fatigue, mood disorders), and a family history of AIG (4). The follow-up UGE with collection of gastric biopsies from patients with potential AIG was performed every year ( ± 6 months) in order to assess the evolution into overt AIG, defined as the appearance of any grade of atrophy (from mild to severe) in the oxyntic mucosa. UGE was performed by expert (>5 years of experience), board-certified, endoscopists by using Olympus gastroscopes. All macroscopic findings at the time of enrollment and at the time of evolution into overt AIG have been reported. Among all, we included only potential AIG patients who subsequently developed overt AIG and with no concomitant autoimmune disorders. This is because we wanted to make sure the increase of IEL in potential AIG was actually due to the presence of PCA rather than generically reflecting a reverberation of the autoimmune diathesis from other diseases (17). Fasting serum 17-gastrin (normal value <98 pg/mL) was also assessed in all potential AIG patients.

As control groups, we also enrolled PCA-negative patients undergoing UGE for other reasons (i.e., dyspepsia and/or gastroesophageal reflux disease) who had normal gastric histology (healthy controls, HC); patients with mild AIG (i.e., corpus atrophy involving <30% of the specimens from the same compartment); patients with severe AIG (i.e., corpus atrophy involving >60% of the specimens from the same compartment). As disease control groups, we included a number of disorders that are known to be characterized by increased IEL stomach infiltration (17), as a consequence of gastric infection (active H. pylori gastritis) or digestive (active CD) and non-digestive (Hashimoto’s thyroiditis) autoimmunity. In particular, 15 HC (median age 56 years, IQR 40-78; 12 females), 22 AIG patients with mild corpus atrophy (median age 65 years, IQR 50-81; 14 females), 21 AIG patients with severe corpus atrophy (median age 61 years, IQR 49-72; 12 females), 15 patients with active H. pylori gastritis (median age 50 years, IQR 46-57; 10 females), 15 patients with active CD (median age 45 years, IQR 32-57; nine females), and nine patients with Hashimoto’s thyroiditis (median age 55 years, IQR 51-63; six females) were included. In all control groups, PCA were tested and were negative.

### Histopathological Assessment

Deep and superficial CD3+ IEL infiltration was assessed through immunohistochemistry in patients with potential AIG, both at the time of the diagnosis and at the time of the evolution into overt AIG, and in all the other control groups. In particular, 4 μm-thick sections of biopsy tissue samples from the gastric body were stained on a Dako Omnis platform with the antibodies CD3 (polyclonal, Dako, Santa Clara, CA) and chromogranin-A (monoclonal, Ventana, Roche, Rotkreuz, Switzerland). CD3+ IEL were separately quantified in 10 series of 100 contiguous gastric epithelial cells and reported as the mean number of IEL per 100 epithelial cells. They were distinguished into deep IEL (i.e., infiltrating the epithelium of oxyntic glands, including both the isthmus-neck region and the base of the gland) and superficial IEL (i.e., infiltrating the surface foveolar epithelium). Importantly, IEL infiltrating the intestinal metaplastic epithelium, when present, were excluded from the counts. Other histopathological alterations were also reported, including acute or chronic inflammation, intestinal or pseudopyloric metaplasia, and neuroendocrine cell hyperplasia. In particular, chromogranin A-positive ECL cell hyperplasia was assessed according to Solcia et al. classification, when present (19). The presence of gastrin cell hyperplasia was also assessed.

AIG diagnosis was made according to the updated Sydney-Houston criteria, evaluating at least five random gastric biopsies with hematoxylin and eosin and Giemsa stains: two in the antrum, two in the body, and one in the angulus (17). Atrophy was defined as the loss of glands appropriate to the gastric compartment, including both glandular loss and metaplastic (pseudopyloric and/or intestinal) transformation of the native glands (20, 21). Patients with uncertain histopathological findings or with atrophic pangastritis were excluded. Three expert gastrointestinal pathologists (AV, OL, GA) reviewed all biopsies. H. pylori infection was assessed by examining gastric biopsies with Giemsa stain, in search of microorganisms morphologically consistent with H. pylori. Additionally, H. pylori negativity was also ascertained by stool antigen test in all potential AIG patients. Finally, according to internationally agreed criteria, CD diagnosis was based on the presence of serum CD-specific antibodies (i.e., anti-tissue transglutaminase IgA and/or anti-endomysial IgA, in the absence of IgA deficiency) and consistent duodenal histological features (i.e., villous atrophy, increased IEL and lamina propria mononuclear cell infiltration, and crypt hyperplasia/hypertrophy) (15).

### Statistical Analysis

Continuous variables were described with the mean and standard deviation or the median and interquartile range (IQR; 25th-75th percentiles), and categorical variables as counts and percentages. As a primary aim, in patients with potential AIG only, a difference of IEL at the time of the first evaluation and at the onset of overt AIG was assessed with Wilcoxon sign ranked test. As a secondary aim, deep and superficial CD3+ IEL were compared among all groups by means of Kruskall Wallis test. Bonferroni correction was applied for *post-hoc* comparisons. Optimal cut-off point, maximizing sensitivity and specificity, for deep and superficial CD3+ IEL discriminating the whole spectrum of AIG from the other control groups, was assessed by means of receiver operating characteristics (ROC) curves, along with their area under curve (AUC). Stata 16 (StataCorp, College Station, TX, USA) was used for all analyses. The study was approved by the local ethics committee (Protocol number: P3599/2017, San Matteo Hospital Foundation, as an extension of a previously accepted protocol covering the study period) and all patients provided written informed consent before taking part in the study for the anonymized use and publication of their data for research purposes, within the respect of their privacy.

## Results

**Figure 1:**
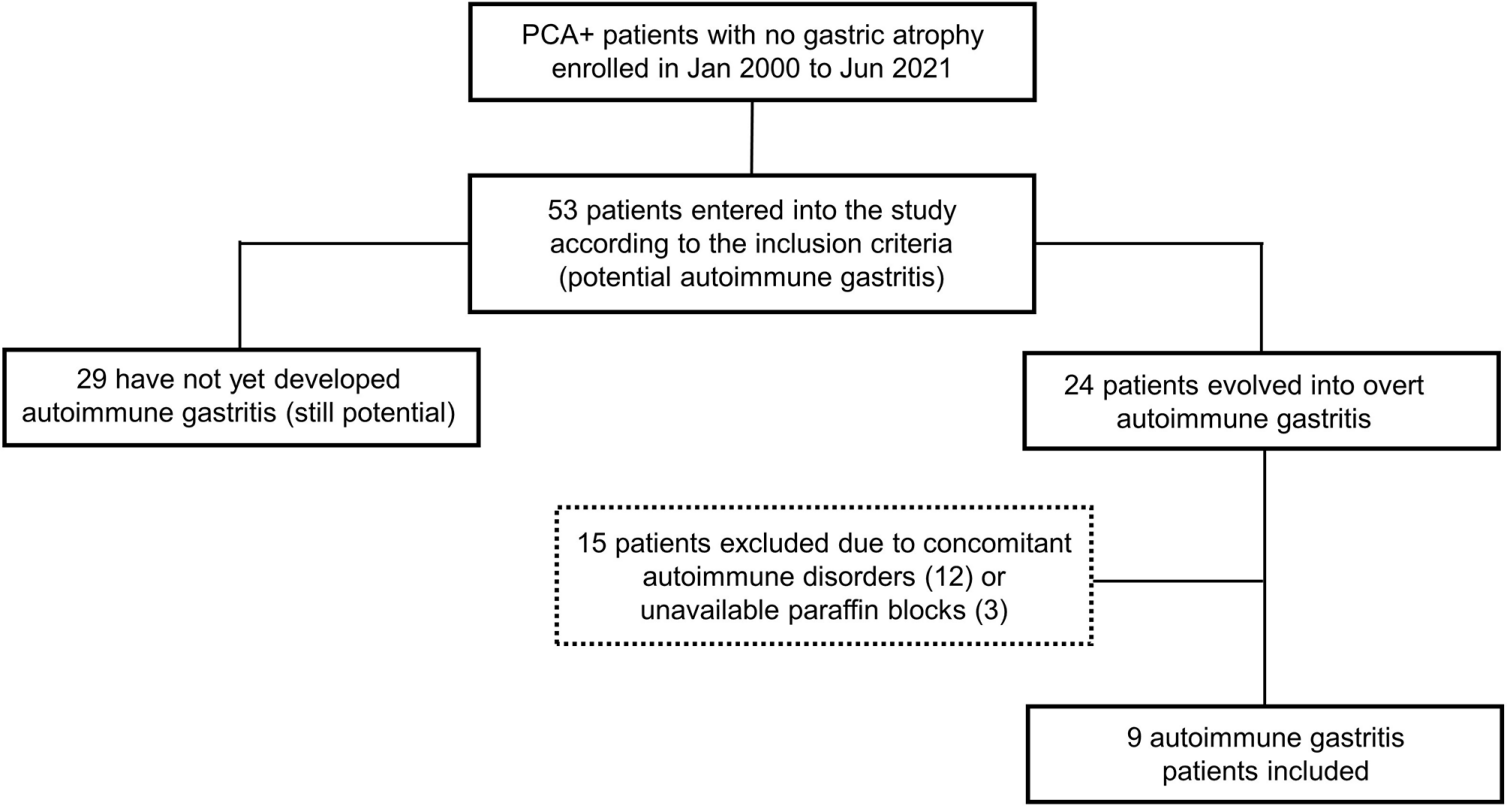
Flowchart showing the selection of patients with potential autoimmune gastritis (AIG). Over the study period, 53 patients turned out to have serum anti-parietal cell antibodies (PCA) and no gastric atrophy. Of these, at follow-up, 24 developed overt AIG, defined as the occurrence of any grade of atrophy in the oxyntic mucosa. For the purposes of the present study, we excluded potential AIG patients with concurrent autoimmune disorders (12 cases), as well as patients in whom the gastric paraffin blocks were not available (3 cases). Hence, nine potential AIG patients were eventually included.

The flowchart of the selection of patients with potential AIG is shown in **Figure 1**. Deep and superficial CD3+ IEL were assessed in the oxyntic mucosa in nine potential AIG patients (median age 61 years, IQR 53-82; four females) before and after the development of gastric atrophy. Of note, deep and superficial CD3+ IEL count did not statistically differ before (median 9.6, IQR 8.8-12.4, and 5.9, IQR 5.0-9.2, respectively; p=0.766) and after (median 11.3, IQR 9.4-12.9, and 7.0, IQR 5.8-11.4, respectively; p=0.514) the onset of overt AIG. At baseline, fasting serum 17-gastrin was within normal range in all nine patients (median 46, IQR 32-59 pg/mL).

**Figure 2:**
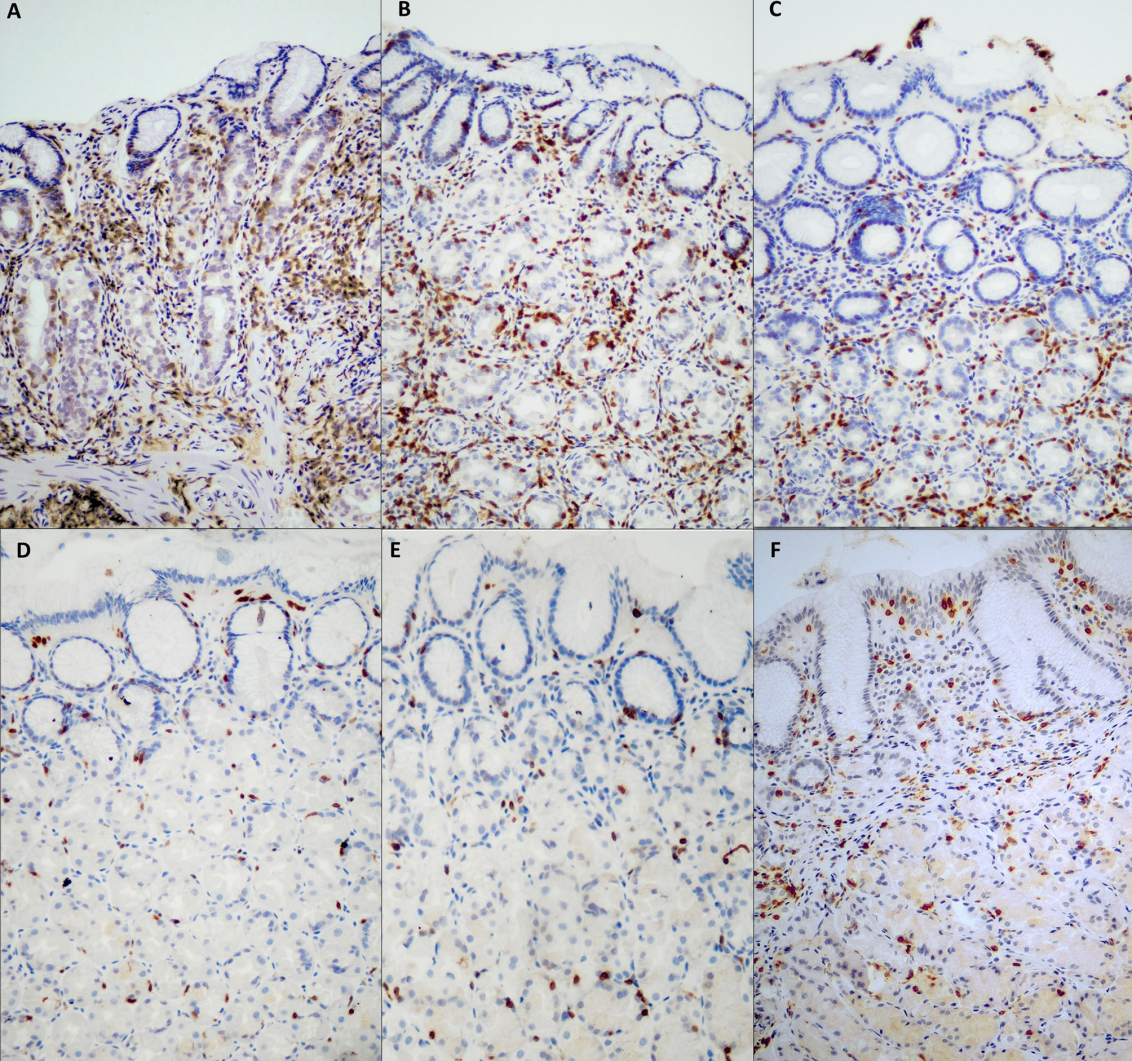
CD3+ intraepithelial lymphocyte (IEL) distribution in samples of oxyntic mucosa (gastric body) from different groups. In severe AIG **(A)**, atrophic oxyntic mucosa is evident, together with a heavy CD3+ IEL infiltration in the deep portion of glands. In potential AIG at diagnosis **(C)**, a high number of CD3+ IEL is evident in deep oxyntic glands, resembling severe AIG **(A)** and mild AIG **(B)**, but in the absence of glandular atrophy. In Hashimoto’s thyroiditis gastric mucosa **(E)**, a low number of both superficial and deep intraepithelial CD3+ lymphocytes are evident, similarly to HC **(D)**. In **(H)** pylori infection **(F)**, CD3+ lymphocytes predominantly infiltrate the surface foveolar epithelium. [CD3 immunohistochemistry; original magnification, 200x]. AIG, autoimmune gastritis; HC, healthy control.

The results of the deep and superficial CD3+ IEL counts are reported in **Table 1**. The overall interobserver agreement among the three pathologists was 100%. Regarding deep CD3+ IEL count, this was significantly higher (p<0.001) at diagnosis of potential AIG, in mild AIG, and in severe AIG compared to both HC and H. pylori gastritis. Regarding superficial CD3+ IEL count, this was significantly higher (p<0.001) in active CD compared to mild AIG and Hashimoto’s thyroiditis, while no significant difference was found between potential AIG and the other groups. Representative images of the oxyntic mucosa (gastric corpus) stained with CD3 by immunohistochemistry in potential, mild, severe AIG, HC, Hashimoto’s thyroiditis, and H. pylori gastritis are shown in **Figure 2**. Of note, patients with potential AIG, mild AIG, and severe AIG all showed a high number of CD3+ IEL in deep glands, while in all the other groups only a few IEL were noticed.

**Table 1:** Deep and superficial CD3+ intraepithelial lymphocyte (IEL) counts (median and interquartile range) in the seven study groups.

	Deep CD3+ IEL	Superficial CD3+ IEL
Healthy controls	4.2 (3.4-5.5)	7.0 (5.4-10.7)
Severe AIG	11.3 (9.3-12.8)	7.9 (5.9-12.2)
Mild AIG	10.3 (6.4-13.2)	6.0 (5.0-7.6)
Potential AIG	9.6 (8.8-12.4)	5.9 (5.0-9.2)
H. pylori gastritis	3.9 (3.1-4.6)	8.6 (5.8-14.9)
Active celiac disease	5.6 (4.8-12.6)	11.8 (10.0-17.4)
Hashimoto’s thyroiditis	6.5 (6.1-8.3)	6.3 (3.2-7.2)
p-value (global)	0.0001	0.0007

AIG, autoimmune gastritis. The adjusted p-value for significance for multiple comparisons between groups was 0.001.

**Figure 3:**
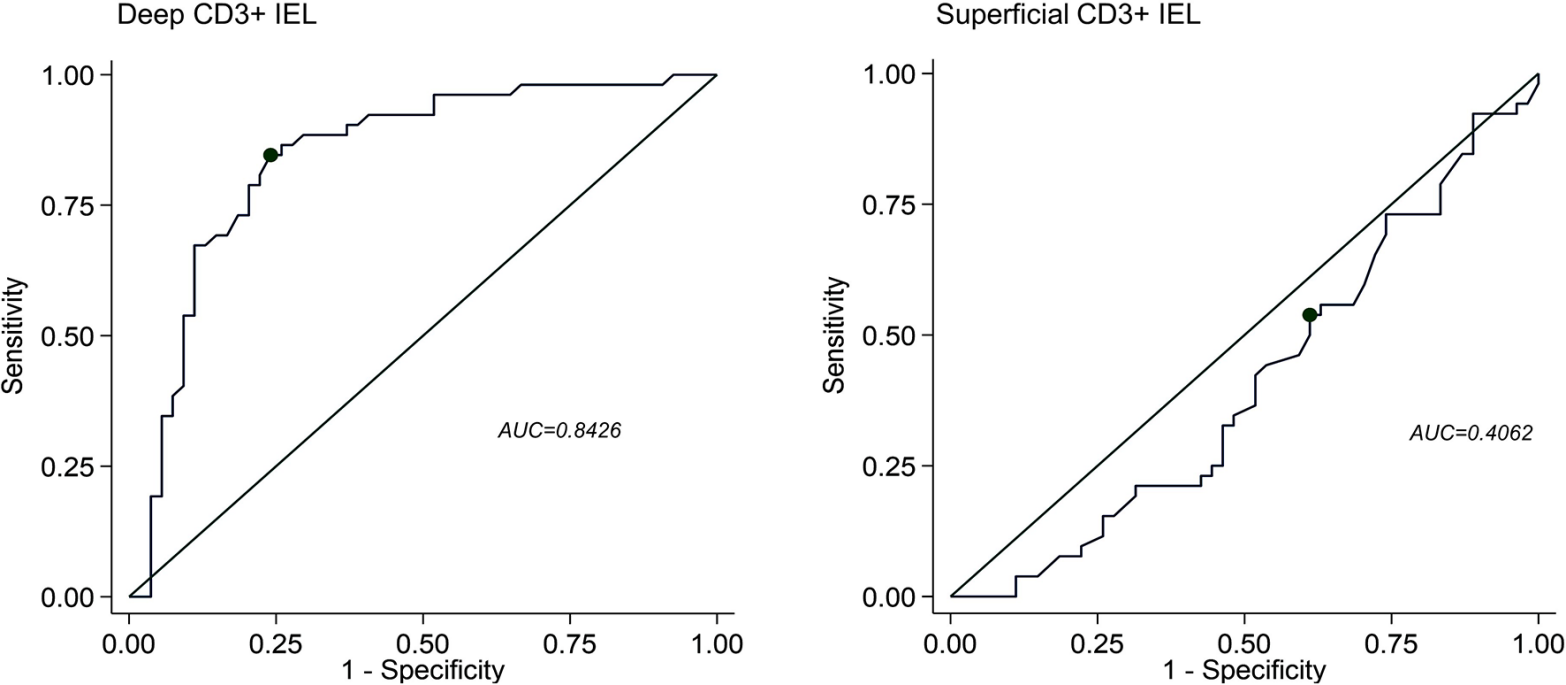
Receiving operating characteristics (ROC) curves discriminating patients with autoimmune gastritis (AIG) compared to healthy controls, H. pylori gastritis, active celiac disease, and Hashimoto’s thyroiditis according to deep (right panel) or superficial (left panel) CD3+ intraepithelial lymphocyte (IEL) infiltration. AUC, area under curve.

The ROC curves discriminating patients with AIG (any stage and severity) compared to HC, H. pylori gastritis, active CD, and Hashimoto’s thyroiditis according to deep or superficial CD3+ IEL counts are shown in **Figure 3**. The superficial CD3+ IEL counts (left side) showed a poor AUC. Instead, the optimal cut-off for deep CD3+ IEL counts was 7/100 epithelial cells (sensitivity 85%, specificity 76%).

At the time of potential AIG diagnosis, the UGE showed mild corpus and antrum erythema in three cases, mild flattening of gastric folds in one, and normal findings in five. At the time of evolution into overt AIG, UGE showed moderate to severe flattening of gastric folds in six patients, mild flattening of gastric folds in two patients, and mild diffuse erythema in one patient. At histopathology, before the development of overt AIG, four patients developed foveolar hyperplasia, while three patients had mild chronic inflammation in the gastric corpus mucosa. Intestinal metaplasia, epithelial or neuroendocrine dysplasia, and gastrin or ECL cell hyperplasia were never noticed before the evolution into overt AIG. At the time of evolution into overt AIG, seven patients had pseudopyloric metaplasia, four patients had intestinal metaplasia, and all nine patients had linear ECL cell hyperplasia in the gastric corpus and gastrin cell hyperplasia in the antrum. None of these nine patients developed a neuroendocrine tumor, nor epithelial dysplasia or neoplasia. Fasting serum 17-gastrin was always increased at the time of evolution into overt AIG (median 178, IQR 159-198 pg/mL).

## Discussion

We have recently described that roughly half of patients with potential AIG evolve into overt AIG in a median time of two years and having at least a concomitant autoimmune disorder dramatically increases the risk of evolution (22). Our present study has provided further novel insights into the definition of the pre-atrophic phase of AIG, the pathological alterations of which had never been described. Before the first definition of potential AIG was formulated (11), a number of pathological features have been described in the early phase (23) of AIG, namely diffuse or focal deep lymphoplasmacytic infiltration within the lamina propria, patchy destruction of individual oxyntic glands, parietal cell pseudohypertrophy, eosinophilic inflammation, intestinal metaplasia of the body and G/ECL cells hyperplasia (24–28). However, some of these features are either unspecific or are now considered as a part of the spectrum of overt AIG (1), as in the case of neuroendocrine cells hyperplasia and intestinal metaplasia which both underlie the presence of atrophy. Similarly, small case series regarding the endoscopic appearance of the stomach in the pre-atrophic phase of AIG have been published, showing either unspecific or very mild alterations (e.g., mucosal erythema, especially of the fundus mucosa, and the presence of hyperplastic polyps) (29), which, however, may also be found in healthy individuals and in other gastric diseases. Hence, at present, there are no specific clinical, endoscopic, and histopathologic features of very early, pre-atrophic AIG. Of note, increased IEL infiltration in the mid and deep gastric glands has also been recently described in a small series of patients who had no features of overt AIG, nor of H. pylori-related gastritis, but were more likely to suffer from other autoimmune diseases (17). Speck et al. hypothesized that at least a subset of those patients might have had “early AIG”, although follow-up biopsies, when available, did not show a progression into overt AIG. Also, in only one patient were PCA detected, while it is unknown whether the other cases were PCA negative or positive. Indeed, while increased IEL infiltration of the gastric mucosa may also be found in other non-gastric, immune-mediated disorders, the presence of circulating PCA is the qualifier of potential AIG.

According to our data, potential AIG patients, in face of an architecturally normal mucosa, had increased deep CD3+ IEL compared to both HC and active H. pylori gastritis, and a IEL cut-off of >7/100 epithelial cells allowed discrimination also from patients with other autoimmune diseases with an overall good accuracy. In patients having this feature, PCA should indeed be tested. Similar to what is seen in the duodenal mucosa of patients with suspected CD, increased IEL infiltration may be considered as nonspecific (30) unless CD-specific autoantibodies are present. The accuracy of PCA in real-life settings has been claimed to be suboptimal (around 70-80%), with a relatively high number of false positivity, and strictly dependent on the detection method (immunofluorescence technique vs ELISA) (31–33). On the contrary, we have shown that these PCA-positive patients should not be labeled as being false positive, but rather should be followed up over time as at least a part of them will develop AIG. Hence, the overall accuracy of PCA could be higher. Starting from these considerations, in the absence of updated guidelines, in patients with potential AIG we would suggest scheduling a UGE with gastric biopsies every 3 to 5 years. In fact, the latest guidelines regarding atrophic gastritis have been recently published and do not include potential AIG (34). Fasting 17-gastrin could also be used as an accurate biomarker to assess serologically the onset of gastric atrophy prior to the follow-up endoscopy. According to a recently published paper, fasting 17-gastrin levels well correlate with the diagnosis of overt AIG and type 1 gastric neuroendocrine tumors (35).

Even if this was not the primary scope of the study, we have also found that patients with active CD had a significantly increased number of superficial CD3+ IEL in the oxyntic mucosa. This is not a novel finding, as a lymphocytic gastritis pattern has already been described in these patients (36). Indeed, this feature should raise the suspicion of CD.

We are aware that our study has some pitfalls and limitations. First, in terms of absolute numbers, the sample size of patients with potential AIG is rather small, and this might cause a serious interpretation bias. This is because our study was monocentric, conducted in a tertiary referral center, and encompassed a rather long time span during which some laboratory (i.e., PCA detection method) and technological (i.e., the use of high-definition endoscopes) advancements have certainly been made. Hence, our data should be considered as preliminary and must be validated in a larger, multicentric, prospectively enrolled, population, and interobserver agreement among pathologists should be assessed in other non-academic settings. Also, a larger study would allow the assessment of all the precocious histopathological alterations, especially in relation to the development of gastric dysplasia, neoplasia, or neuroendocrine tumors. A deeper analysis of the risk-benefit balance of UGE reassessment should be made before drafting *ad hoc* guidelines. Nonetheless, our study has some strengths and novel findings that should be highlighted. This is the first prospective study that demonstrates that PCA, along with increased deep CD3+ IEL infiltration, is a marker of AIG in its pre-atrophic stage. In order to avoid possible confounding factors, we excluded patients with potential AIG and concurrent Hashimoto’s thyroiditis and CD. Also, we here delineated a possible congruous endoscopic follow-up for these patients.

To conclude, we herein found that an increased number of deep CD3+ IEL in the oxyntic mucosa (>7/100 epithelial cells, as per optimal cut-off identified by the ROC curve) without concomitant metaplasia, atrophy, or inflammation of the lamina propria represents a histopathological marker of potential AIG, when serum PCA are present. We, therefore, urge for a better characterization of patients with potential AIG in order to prevent potentially life-threatening complications, such as vitamin B12 deficiency-related alterations (i.e., pernicious anemia, irreversible neuropathy), neuroendocrine tumors, and gastric adenocarcinoma. In addition, studies assessing the accuracy of PCA–in the light of our results are eagerly awaited.

## Data Availability Statement

The original contributions presented in the study are included in the article/supplementary material, further inquiries can be directed to the corresponding author.

## Ethics Statement

The studies involving human participants were reviewed and approved by San Matteo Hospital Foundation. The patients/participants provided their written informed consent to participate in this study.

## Author Contributions

ADS, MVL, EM, and AV designed the study. EM, MVL, and ADS enrolled and followed-up patients, collected and analyzed data. CK made and interpreted all statistical analyses. ML collated, interpreted data, and wrote the manuscript. AV, FC, and GA reviewed all histopathological specimens. ADS, CK, GRC, EM, and MVL made critical revision of the manuscript for important intellectual contents. All other authors enrolled patients, collected data and reviewed the paper. All authors significantly participated in the drafting of the manuscript or critical revision of the manuscript for important intellectual content and provided approval of the final submitted version.

## Funding

We thank the “Rete Aging” project - Italian Ministry of Health, for supporting research focusing on diseases and conditions affecting the elderly.

## Conflict of Interest

The authors declare that the research was conducted in the absence of any commercial or financial relationships that could be construed as a potential conflict of interest.

## Publisher’s Note

All claims expressed in this article are solely those of the authors and do not necessarily represent those of their affiliated organizations, or those of the publisher, the editors and the reviewers. Any product that may be evaluated in this article, or claim that may be made by its manufacturer, is not guaranteed or endorsed by the publisher.
